# Improving turnaround times with artificial intelligence in microbiology

**DOI:** 10.1128/jcm.00354-26

**Published:** 2026-07-10

**Authors:** Ross Davidson, Charles Heinstein, Glenn Patriquin, Lee William Goneau, Leah Ashleigh Brown, Brian James Hill

**Affiliations:** 1 Division of Microbiology, Department of Pathology, Queen Elizabeth II Health Sciences Centre 3686, Halifax, Nova Scotia, Canada; 2 Department of Pathology, Dalhousie University 3688, Halifax, Nova Scotia, Canada; 3 Dynacare Laboratory, Brampton, Ontario, Canada; 4 Department of Laboratory Medicine, University of Toronto 7938, Toronto, Ontario, Canada; 5 Copan Diagnostics, Inc., Murrieta, California, USA; Mayo Clinic, Rochester, Minnesota, USA

**Keywords:** UTI, artificial intelligence, automation

## Abstract

This dual-center study evaluated the impact of artificial intelligence (AI) on urine culture turnaround times in Canadian diagnostic laboratories using microbiology laboratory automation. Data were collected before and after the implementation of PhenoMATRIX (PM), an AI-based software that provides continuous culture sorting and result interpretation support. In both a low-volume tertiary care hospital and a high-volume community laboratory, PM enabled earlier availability of interpretable results; however, reductions in time to result reporting (TTRR) were contingent on workflows for result release. At the tertiary care site, implementation of PM alone was associated with increased TTRR, reflecting delays between result availability and reporting. Integration of PM+ enabled automated release of negative results as they became available, resulting in a TTRR reduction of approximately 1.3 h. At the community laboratory, where PM+ was not implemented, a TTRR improvement of approximately 5.3 h was achieved by advancing manual screening workflows (08:00 vs 16:00), facilitating earlier review and release of PM-generated results. These findings indicate that AI-driven culture assessment reduces TTRR when coupled with processes that enable timely result release, either through automated reporting or optimized laboratory review workflows.

**IMPORTANCE:**

Advances in artificial intelligence (AI), coupled with the power of laboratory automation, is the next step in the evolution of the clinical microbiology laboratory. We demonstrated that bacterial culture assessment (urine cultures) by AI decreases the time required to release results to clinicians and reduces the hands-on time required by technologists to analyze and finalize laboratory analysis. This study was performed in two different laboratories that differed not only in geography but also in scope and scale. The same ultimate benefits were seen in both institutions demonstrating that this technology is widely applicable for most laboratories.

## INTRODUCTION

Automation in clinical microbiology has become essential to address persistent staffing shortages and increasing specimen volumes. Automated technologies improve efficiency compared to manual methods across laboratories of varying size and patient acuity (1). Following implementation of automation, artificial intelligence (AI) algorithms offer the next step in optimizing workflows.

Copan’s PhenoMATRIX (PM) software (Copan Diagnostics, Murrieta, CA 92652, USA) uses AI algorithms to continuously assess and sort culture plates based on software interpretations of bacterial growth from WASPLab-generated digital plate images. For urine cultures, the software categorizes plates demonstrating no growth, no significant growth (<10 colonies/plate), mixed growth (>2 colony types), and/or specific colony colorations on chromogenic media. Prior studies have shown the accuracy of these algorithms across multiple specimen types and media (2–6). Beyond accuracy, AI has the potential to accelerate result reporting through continuous culture assessment.

This study evaluated time to result reporting (TTRR) using PM in a dual-center Canadian study of urine cultures. Additionally, PhenoMATRIX PLUS (PM+) expands on PM by enabling automatic result release based on lab-defined criteria without staff involvement, as evaluated at one site. This represents the first dual-site evaluation of PM AI-driven algorithms and their impact on TTRR in North America.

## MATERIALS AND METHODS

This study was conducted at two Canadian diagnostic laboratories with differing volumes, patient populations, and geographic settings. The first site, Queen Elizabeth II Health Sciences Centre (QEII), is a large tertiary care hospital located in Halifax and provides 24/7 diagnostic testing on behalf of the Nova Scotia Health Authority for other laboratories across the province. The laboratory also serves as the reference laboratory for the Canadian Atlantic region, processing over 65,000 urine specimens annually. The QEII is staffed with medical lab technologists from 7 a.m. to 11 p.m. and with a medical laboratory assistant from 11 p.m. to 7 a.m. The second site, Dynacare Laboratories in Brampton, Ontario, is a high-volume community laboratory operating 24/7 and processing an annual average of approximately 650,000 urine specimens collected across the province.

The study was conducted over a 3-year period from May 2022 to May 2025, with discrete data collection timepoints at each site before and after PM and PM+ implementation. At QEII, TTRR data were collected in June 2023 (PrePM), September 2024 (PostPM), and May 2025 (PostPM+). At Dynacare, data were collected in May 2022 (PrePM) and May 2023 (Post-PM).

For Dynacare, PrePM and PostPM TTRR were defined as the time from specimen placement on the WASPLab (Copan Diagnostics) to final approval and release of the culture report in the laboratory information system (LIS). At QEII, TTRR was calculated from specimen receipt in the laboratory to result release in the LIS. At both laboratories, TTRR included both organism identification (ID) and/or antimicrobial susceptibility test (AST) reporting when performed.

Midstream urine specimens were plated and incubated using WASPLab automation and imaged at approximately 16 h of incubation. At both Dynacare and QEII, organism identification was performed using Vitek MS, and antimicrobial susceptibility testing (AST) was conducted by MALDI-TOF using Vitek 2 MS (MS) in accordance with CLSI guidelines. At Dynacare, *Escherichia coli* exhibiting characteristic pink colonies on Oxoid Brilliance UTI Clarity Agar was reported without MS confirmation. QEII utilized chromogenic Orientation Agar for primary culture and MS for culture identification. *E. coli* exhibiting characteristic colonies on Orientation Agar were reported without MS confirmation.

Workflow changes occurred at Dynacare following PM implementation to facilitate review and release of PM-generated results, which were available earlier than the previously manually interpreted cultures PrePM. Urine culture screening expanded from evening-only hours (16:00–24:00) PrePM to combined day and evening shifts (08:00–24:00) PostPM. In addition, one full-time equivalent (FTE) was reallocated from the urine bench to another area following PM implementation due to efficiency gains. These changes were considered in the interpretation of study outcomes.

At QEII, urine cultures were screened on the day shift (07:00–16:00) for all TTRR timepoints measured (PrePM, PostPM, and PM+). Urine cultures with a final result of no growth or normal urogenital flora of less than 10 colonies were automatically released and reported via PM+.

## RESULTS

### QEII

**Fig 1:**
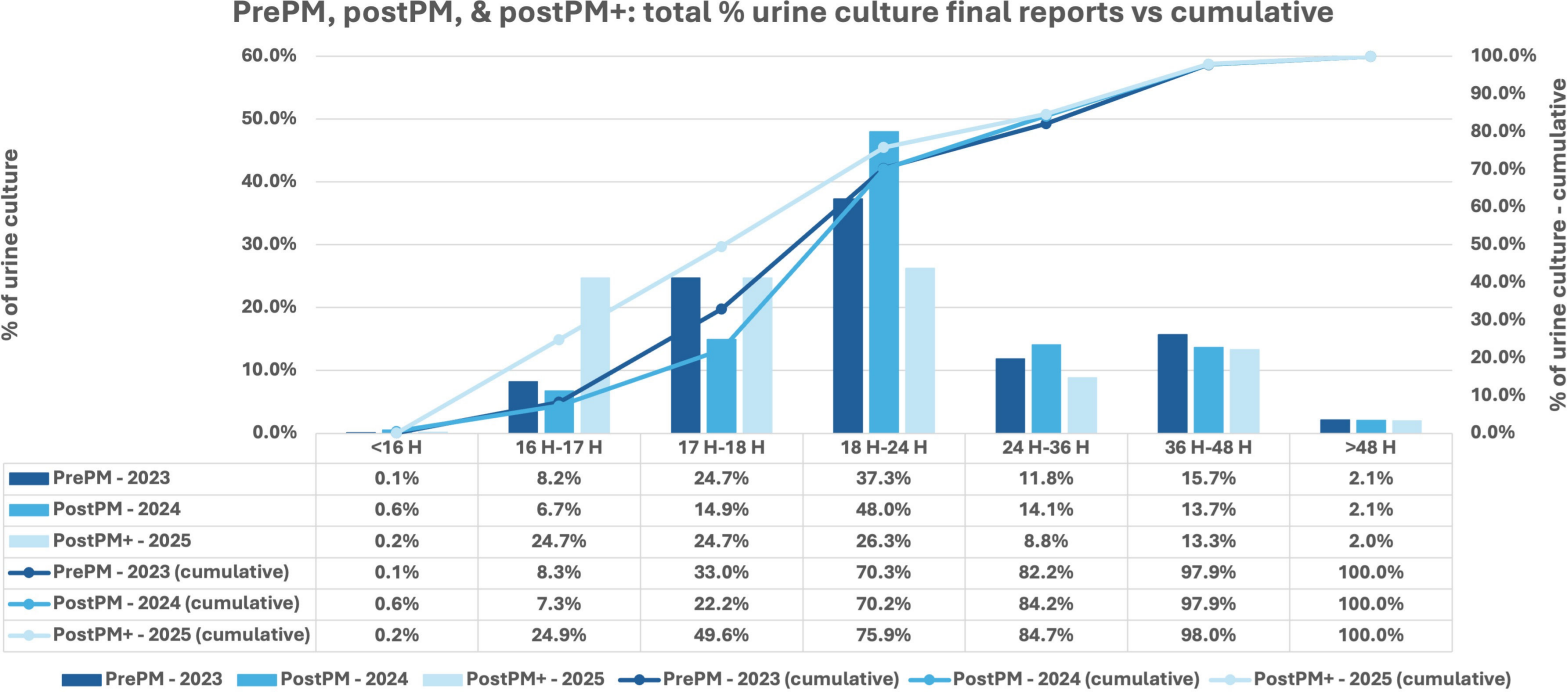
TTRR for PrePM, PostPM, and PostPM+ final culture reports; QEII.

During the study period, the daily average of urine cultures increased from 167 in 2023 to approximately 180 in 2024–2025, representing a ~7% vol increase. The TTRR for PrePM, PostPM, and PostPM+ phases are shown in Fig. 1. The PrePM urine cultures were reported as negative, positive, and mixed growth in 56%, 23%, and 21% of cases, respectively, compared to 54%, 24%, and 22% for the PostPM phase and 58%, 25%, and 17% for the PostPM+ phase.

With the implementation of PM+, the greatest shift in TTRR occurred within the first 2 h after the 16-h image was taken. Specifically, the percentage of final reports released by 17 h increased to 24.9%, compared with 8.3% in the PrePM phase and 7.3% in PostPM. By 18 h, 49.6% of urine cultures had a final report available, compared with 33.0% (PrePM) and 22.2% (PostPM). At 24 h, 75.9% of final urine culture results were released with PM+, compared with 70.3% (PrePM) and 70.2% (PostPM). Approximately 77% of the QEII’s urine cultures are negative or reported as normal urogenital flora. Consequently, the proportion of cultures pending final reports beyond 24 h decreased from 29.7% (PrePM) and 29.8% (PostPM) to 24.1% (PM+). Overall, the mean TTRR decreased by approximately 1 h and 19 min between June 2023 and May 2025.

### Dynacare

**Fig 2:**
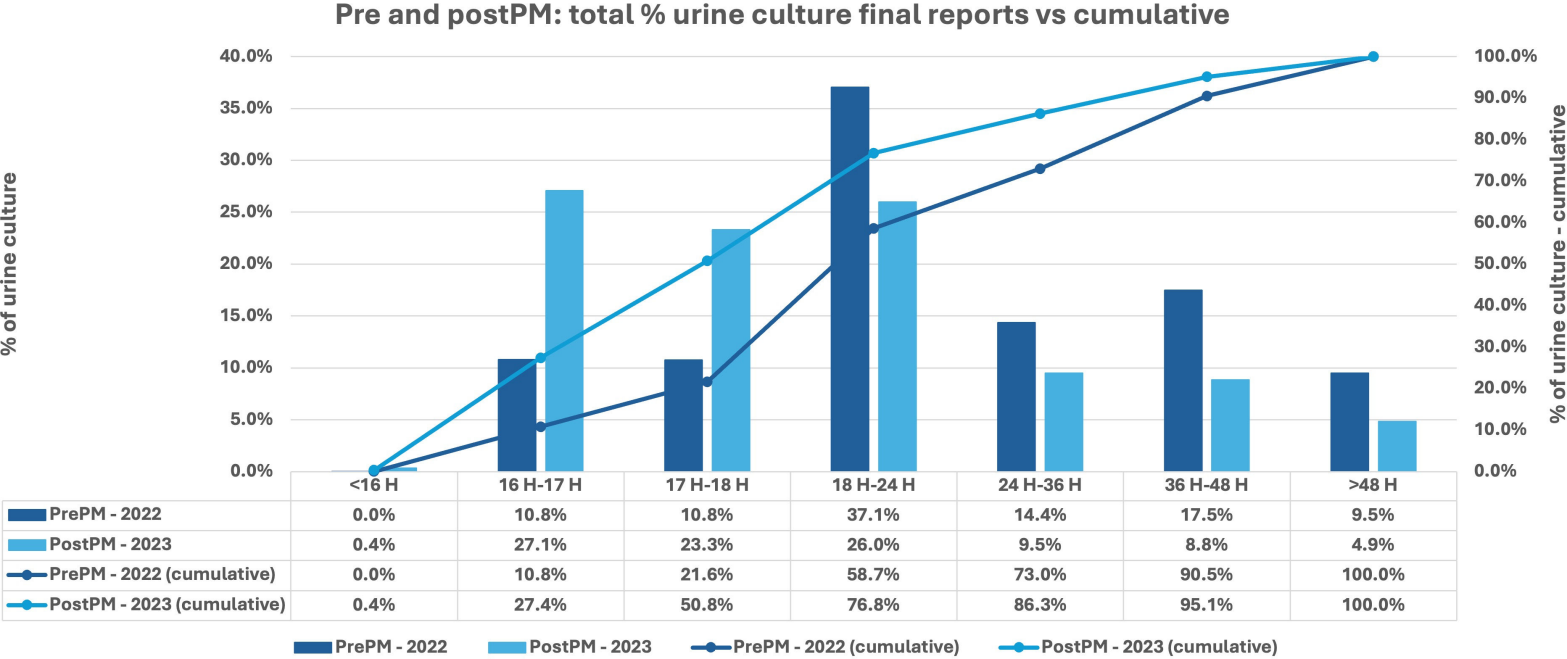
TTRR results for PrePM and PostPM final culture reports: Dynacare.

At Dynacare, the average daily urine culture volume increased from 1,593 in 2022 to 1,778 in 2023 (~11.6% vol increase). In May 2022 (PrePM), urine cultures were reported as negative, positive, and mixed growth in 53%, 29%, and 18% of cases, respectively, compared to 51%, 29%, and 20% in May 2023 (PostPM). TTRR for PrePM and PostPM phases are shown in Fig. 2.

Following PM implementation, a greater proportion of final reports were released within 18 h compared to those released after 18 h. The proportion of final culture reports released by 17 h increased from 10.8% (PrePM) to 27.4% (PostPM). By 18 h, 50.8% of reports were finalized compared to 21.6% before implementation, and by 24 h, 76.8% of final urine culture reports were released vs 58.7% PrePM. The proportion of final culture reports pending beyond 24 h decreased by 18.1% (41.3% PrePM to 23.2% PostPM). Overall, the mean final report TTRR improved by approximately 5 h and 18 min from May 2022 to May 2023.

A small proportion of urine cultures (0.4%) in the PostPM data set were finalized before 16 h of incubation. Case review showed these reflected either administrative outcomes (e.g., duplicate specimen cancelations or specimens not processed due to laboratory incidents) or cultures imaged slightly earlier (~15.5 h) by WASPLab and finalized promptly when no ID or AST was required (e.g., no growth). These instances reflect minor timing variation related to automated imaging schedules rather than systematic deviation from incubation protocols.

## DISCUSSION

This dual-site study demonstrates that artificial intelligence-assisted culture interpretation improves TTRR in automated microbiology laboratories; however, the magnitude of improvement depends on workflows that enable timely result release. While PM facilitated earlier availability of interpretable culture results at both sites, reductions in TTRR were only realized when paired with processes that supported earlier review or automated reporting.

At the tertiary care site (QEII), implementation of PM alone did not reduce TTRR, reflecting delays between result availability and manual release. In contrast, integration of PM+, which enabled automated release of negative and non-significant cultures, resulted in a measurable reduction in TTRR (~1.3 h) and a shift toward earlier reporting timepoints. This finding highlights that AI-driven interpretation improves efficiency primarily when coupled with mechanisms that translate earlier result availability into earlier reporting.

At the high-volume community laboratory (Dynacare), where PM+ was not implemented, a larger reduction in TTRR (~5.3 h) was observed following PM implementation. This improvement was not solely attributable to AI interpretation, but rather to concurrent workflow modifications that enabled earlier result review, including expansion of urine culture screening from evening-only to combined day and evening shifts. These changes facilitated earlier action on PM-generated results and underscore the importance of aligning laboratory workflows with AI-enabled data availability.

In addition to improvements in TTRR, implementation of PM at Dynacare was associated with measurable productivity gains, including the reallocation of one full-time equivalent (FTE) technologist from the urine bench. When normalized to test volume, this represents a reduction in technologist time per specimen, reflecting increased efficiency in culture screening and result interpretation. These findings support the role of AI not only in accelerating reporting but also in optimizing labor utilization in high-throughput environments. In Halifax, the implementation of TLA and PostPM+ allowed the reallocation of two FTE positions. One FTE savings (reallocation) was achieved through a reduction in labor requirements with the introduction of the TLA, and a second FTE was reallocated from the urine screening to another area of the laboratory.

These observations are consistent with prior studies demonstrating that algorithm-assisted culture interpretation (by Culbreath et al.) accelerates reporting and reduces the proportion of cultures requiring extended review (1). The present study extends these findings by demonstrating that the impact of AI on TTRR is not inherent to the technology itself but is contingent on the integration of AI outputs into laboratory workflows.

The ongoing shortage of qualified medical laboratory technologists and increasing specimen volumes underscore the importance of technologies that optimize efficiency without compromising quality. Full laboratory automation has already been shown to improve culture processing and mitigate staffing challenges (7–9). The addition of AI software such as PhenoMATRIX enables continuous assessment of culture plates and prioritization of review. However, as demonstrated in this study, the benefits of AI are maximized when paired with workflow strategies that enable timely release of results, either through automated reporting (PM+) or optimized manual review processes.

The combined benefits of continuous incubation, automated imaging, AI-assisted interpretation (PM), and automated result release (PM+) represent a major advancement in clinical microbiology. Smart incubation has been shown to shorten incubation times while maintaining accuracy and enabling faster identification and antimicrobial susceptibility testing results (10). Future innovations, such as AI-assisted selection of isolates for automated identification and susceptibility testing, could enable end-to-end automation of uncomplicated cultures. This evolution will allow laboratory professionals to focus their expertise on clinically complex specimens while maintaining high throughput and timely patient results.

This study represents, to our knowledge, the first dual-site evaluation in North America assessing the impact of PM AI-driven algorithms on urine culture TTRR. Across both laboratory settings, AI integration into automated microbiology workflows improved the timeliness of result availability, while reductions in TTRR were achieved when supported by workflow changes that enabled earlier reporting. These findings highlight that successful implementation of AI in clinical microbiology depends not only on algorithm performance but also on thoughtful integration into laboratory operations to realize its full clinical and operational benefits.
